# How might diabetes organisations address diabetes‐related stigma? Findings from a deliberative democratic case study

**DOI:** 10.1111/hex.13398

**Published:** 2021-12-02

**Authors:** Heath Pillen, Paul R. Ward

**Affiliations:** ^1^ Discipline of Public Health Flinders University College of Medicine and Public Health Bedford Park South Australia Australia

**Keywords:** community participation, diabetes mellitus, public health practice, social stigma

## Abstract

**Introduction:**

This study sought to identify how diabetes organisations conceptualize the problem of diabetes‐related stigma and how this shapes the selection of stigma‐reduction interventions.

**Methods:**

A qualitative deliberative democratic methodology was used to access an informed dialogue about what should be done by diabetes organisations to address diabetes‐related stigma, drawing from the perspectives of board members, healthcare services staff, and communications and marketing staff from a single state‐wide diabetes organisation in Australia (*n* = 25).

**Results:**

Participants navigated the stigma concept along two axes: one that drew attention to either disease attributes or personal moral attributes as the object of stigmatisation, and one that positioned stigma as an individual or structural problem. This shaped the selection of stigma‐reduction interventions, which included interventions to: (i) reduce the prevalence of stigmatized attributes, (ii) correct misunderstandings about diabetes, (iii) modify representations of persons with diabetes, (iii) enhance coping amongst persons with diabetes and (iv) make healthcare more person‐centred and democratic.

**Conclusion:**

This study identified several grievances with ‘diabetes‐related stigma’, which are grievances that can be conceptualized and addressed at both individual and structural levels, and involve correcting misinformation about diabetes or challenging and communicating alternative representations of persons living with diabetes.

**Patient or Public Contribution:**

The organisation's management and board were consulted throughout all stages of research development, analysis and reporting. The information and vignettes presented to participants drew from illness narratives obtained from earlier research involving adults with type 2 diabetes. Research participants included adults with various diabetes types.

## 1 INTRODUCTION

Stigmatized populations represent particularly vulnerable groups within healthcare systems. They are vulnerable in the sense that perceptions of stigma (self‐ and anticipated‐stigma) contribute to poorer psychological, social and health outcomes^1^, ^2^, ^3^ and may result in the avoidance of healthcare services.^4^ They are also vulnerable in the sense that stigmatisation is intricately bound‐up with social inequality whereby stigmatisation partly emerges from and reproduces existing social inequalities.^5^, ^6^, ^7^ In light of these concerns, better understanding and reducing health‐related stigma has assumed a prominent position within health policy and research. This is especially true of diabetes‐related stigma, which has emerged as a policy issue for the International Diabetes Federation^8^ and is an important area of enquiry for research seeking to improve the mental health and wellbeing of persons with diabetes.^9^, ^10^ While diabetes‐related stigma has historically been seen as less important than the stigma experienced by persons living with disability, mental illness and human immunodeficiency virus–acquired immunodeficiency syndrome (HIV‐AIDS),^11^ there is a growing literature that recognizes the pervasive impact that stigma has on the lives of persons living with diabetes,^1^, ^3^, ^12^, ^13^, ^14^ a point that has been explicitly made by those living with diabetes.^15^

However, there are two key issues confronting stigma‐reduction work within healthcare and public health organisations, both in relation to diabetes and other stigmatized conditions. The first is that there is limited evidence of effectiveness for stigma‐reduction interventions.^11^, ^16^, ^17^ In their review of diabetes‐stigma research in 2013, Schabert et al.^11^ were ‘unable to identify any literature regarding strategies to reduce, or assist people to cope with, diabetes‐related stigma’. More recently, this paucity of evidence has been recognized in the 2019 Diabetes UK position statement on *transforming the mental well‐being for people with diabetes*, which declared a need to identify ‘interventions designed to reduce stigma, learning from existing successful stigma reduction interventions for other stigmatized conditions’.^9^ The second issue is that stigma‐reduction work tends to draw upon fractured understandings of health‐related stigma, which produces a ‘mixed bag of anti‐stigma interventions’^7^ that limits the realisation of stigma‐reduction on a population scale. This fractured understanding of the stigma concept, reflective of the diverse disciplinary and theoretical perspectives used to explain stigmatisation,^18^ means that organisations involved in stigma‐reduction work are likely to require some guidance to navigate the conceptual morass.

The present study seeks to address this second issue by qualitatively mapping how members of a diabetes organisation perceive their role in stigma‐reduction work, and what this infers about how they conceptualize diabetes‐related stigma. Although existing research has attempted to categorize stigma‐reduction interventions,^16^, ^17^, ^19^ no study has attempted to examine how these categories relate to different ways of understanding health‐related stigma from the standpoint of those involved in stigma‐reduction work. Examining stigma reduction from the standpoint of diabetes organisations is important given that diabetes patient advocacy groups and communities have a long history of engaging in stigma‐reduction work. For example, diabetes organisations have historically approached stigma‐reduction work by correcting ‘myths’ and ‘misconceptions’ about diabetes,^8^, ^20^ using high‐profile individuals to communicate the needs and rights of persons living with diabetes,^8^ and through political lobbying to address issues raised by those with diabetes, including issues related to inequities in insurance coverage, access to pharmaceuticals, driver's license standards and discrimination.^20^, ^21^

In the present study, we draw on the findings from deliberative democratic research performed with board members, healthcare services staff and communications and marketing staff from a single Australian state‐wide diabetes organisation. The research specifically sought to identify how staff and board members from this diabetes organisation currently conceptualize the problem of diabetes‐related stigma and how these conceptual understandings are used to justify existing stigma‐reduction work or envisage future approaches to stigma reduction. For diabetes and other patient organisations, this knowledge is valuable because it will allow them to select and communicate a clear stance on stigmatisation, informed by a coherent understanding of health‐related stigma. Doing so will allow patient organisations to more clearly and explicitly ‘identify [their] injuries’ and ‘articulate [their] grievances’,^22^ something that the social disability movement has excelled at in challenging the medicalisation and stigmatisation of impaired persons. At current, there is a limited sense of precisely what grievances might be raised given feelings of being ‘judged, blamed, and shamed’^10^ because of one's diabetes.

## 2 MATERIALS AND METHODS

### 2.1 Research design

This study adopted a qualitative deliberative democratic methodology to help understand how members of a diabetes organisation construct ideas about stigma‐reduction work. Within this approach, different publics are given the opportunity to participate in policy decisions based on an informed and careful (i.e., deliberate) consideration of the issues involved.^23^ In relation to the aims of the present study, this methodology offers advantages over interviews and focus groups in two main ways. First, it purposefully uses participant dialogue as a means of explicating and critiquing the reasoning that informs the selection of a given policy option.^24^ Second, deliberative methodologies accommodate the provision of the policy or other information to participants to promote a more informed public discussion.^25^ Although publics may struggle to *freely* voice their opinions because of the constraining effects of ideology, operating through understandings of the stigma concept,^26^, ^27^, ^28^ the provision of novel information may help ‘individuals see the existing reality in a different light’.^29^ The following discussion of the method expands on how these deliberative elements were integrated into the research.

### 2.2 Participant sampling and recruitment

The organisation (case) examined in this study is an Australian state‐wide, not‐for‐profit and member‐based diabetes organisation that had previously contributed funding to earlier research (2018–2020) examining the role of critical pedagogy in developing an understanding of diabetes‐related stigma amongst adults with type 2 diabetes mellitus (T2DM; manuscript in preparation). Because critical pedagogy seeks engagement between marginalized groups and larger social organisations,^30^ the present study offered an opportunity to bridge the gap between critical qualitative research and social change. The organisation's management and board both agreed in principle to the broad aims and methodology of the research and were consulted during the process of developing a more detailed research protocol. Research participants included members of the organisation's governing board (BRD), healthcare services staff (HCS) and communications and marketing staff (COM). Inclusion of these groups in the research meant that it was possible to explore possibilities for stigma‐reduction intervention across different operational contexts, allowing the research to identify interventions that span public health stigma‐reduction activities of contact, education and advocacy.^19^

Before their involvement in the deliberative research, all staff and board members were provided with a 30‐min presentation (by A1) describing a conceptual model of stigma‐deviance relations for adults with T2DM, which was developed through the aforementioned research (manuscript in preparation). In presenting the conceptual model to participants in the study, the researcher described the way that feelings of shame (in relation to the diseased‐disfigured and/or obese body) and attributions of blame (in relation to failures against standards of bodily self‐care) regarding one's diabetes combine and emerge from dominant ways of knowing about oneself as (medicalized) biological citizens.^31^ The researcher then explained how these ways of knowing are privileged given the assumed ‘riskiness’ of ‘diabetic’ bodies and persons and communicated via news media, health education, health surveillance practices and interpersonal interactions. At the end of the presentation, all persons were offered an information and consent form for the deliberative research. Consent forms were completed and returned before the deliberative groups. Research ethics approval for this project was obtained from the Flinders University Social and Behavioural Research Ethics Committee (Project Number 7899).

### 2.3 Process of deliberation

Four 2‐h‐long deliberative groups (see Table 1 for an overview of participant characteristics) were performed 1–2 weeks following the initial presentation of findings. With the exception of one individual that withdrew due to illness, all persons that attended the information sessions also participated in the deliberative groups. Participants were grouped according to their role within the organisation (as providing governance [BRD], healthcare and preventative health services [HCS], or marketing and communication services [COM]), with HCS staff randomly allocated into one of two groups given the larger size of this cohort. This grouping strategy was designed to help focus group discussion around stigma‐reduction strategies relevant for each role (characteristic of homogenous group sampling) while also ensuring access to a diversity of ideas regarding stigma reduction approaches (characteristic of maximum variation sampling).^32^

**Table 1:** Group composition and participant characteristics

Deliberative group	Relationship to diabetes (BRD)/organisational role (HCS1, HSC2, and COM)	Length of service (years)	Gender
Governing board (BRD)	Family member of person with type 1 diabetes mellitus	<1	Male
	Type 1 diabetes mellitus	3.5	Male
	Not specified	<1	Female
	Type 1 diabetes mellitus	7	Female
	Type 2 diabetes mellitus	10	Male
	Gestational diabetes mellitus (historical)	15.5	Female
Healthcare services staff—Group 1 (HCS1)	Diabetes Educator	4.5	Female
	Executive Manager, Program Development & Delivery	9	Female
	Dietitian/Diabetes Educator	<1	Female
	Dietitian	<1	Female
	Project Officer	<1	Female
	Dietitian—Priority Areas	<1	Female
	Research Trial Coordinator	<1	Female
Healthcare services staff—Group 2 (HCS2)	Evaluation Officer	<1	Female
	Project Officer	<1	Male
	Project Officer—Diabetes Management	2.5	Female
	Health and Service Delivery Manager	4	Female
	Diabetes Educator	<1	Female
	Dietitian	<1	Female
	Diabetes Educator	<1	Female
Communications and marketing staff (COM)	NDSS Training and Assessment Officer	7	Female
	Executive Manager Corporate Services	9	Male
	Member and Community Partnerships Manager	6	Male
	Membership Coordinator	9.5	Male
	Marketing and Communications Officer	5.5	Female

Facilitation was performed by this paper's author (H. P.), with assistance provided by a second postgraduate researcher experienced in qualitative and deliberative research methods. Three short vignettes were firstly presented to each group, based on data excerpts obtained from the aforementioned case study of eight adults with T2DM who had experienced stigmatisation, all of whom were residing in areas serviced by the diabetes organisation within this study. These vignettes functioned to provide a shared text that could focus discussion on different processes involved in stigmatisation while also allowing participants the freedom to interpret, communicate and critique the text in different ways. This approach allowed insight into the norms guiding participants interpretation of the stigmatizing event and ideas about future action.^33^

Vignette 1. I always hear on the news about how the obesity epidemic is resulting in more cases of diabetes and how diabetes is such a burden on the healthcare system. Plus everyone has a story about someone with diabetes that doesn't watch what they eat, ignores their diabetes, and has lost a foot. It's all so tragic. Sometimes it's hard to remain positive about my diabetes.

Vignette 2. I can't say that anyone has treated me cruelly because of my diabetes. But it does annoy me that others feel the need to watch and comment on what I should or shouldn't be eating. It makes me feel like a naughty child for eating the wrong things. I know they probably mean well, but it's none of their business what I eat—the decision and responsibility is solely mine. I mean, we already know our defects, we don't need them pointed out to us.

Vignette 3. I used to feel more shameful about my diabetes before I lost the weight. My old GP used to put everything down to my weight, and all the dietitians I went to all just said I needed to eat less and exercise more. Their attitude was that I just wasn't trying hard enough.

After listening to each vignette, group members were asked to specify what should be done about the situation from the standpoint of a diabetes organisation and offer a rationale for their decision. The role of the facilitator was to keep the discussion on track and moving forward, to facilitate participation, and encourage participants to expand upon, evaluate, and critique the proposed actions and reasoning offered by other participants.

### 2.4 Data collection and analysis

Group discussions were recorded using a digital audio recorder and then professionally transcribed. NVivo qualitative research software (QSR International Pty Ltd, Version 11) was used to organize and support the analysis of all collected data. Data analysis firstly involved a single analyst (H. P.) scanning the transcripts to identify proposed actions that a diabetes organisation might take to reduce diabetes‐related stigma, which were then organized under the typology of stigma‐mitigation approaches described by Weiss et al.^34^ (i.e., addressing the health problem, stigmatizers, emotional impact of stigmatisation and social policy). To ensure that the analysis would build upon existing conceptual knowledge about health‐related stigma and stigma reduction work while allowing for novel insights to be produced, we followed an analytical process involving stages of precoding, conceptual and thematic conceptualisation, and theoretical categorisation.^35^ Using this approach, the analyst (H. P.) firstly used analytic induction to code and categorize different forms of reasoning used to justify each stigma‐reduction intervention, including deliberative critiques and counter arguments. Using abductive and retroductive forms of reasoning (relating to theoretical categorisation), the same author then identified what these different intervention‐reasoning configurations inferred about the stigma‐concept, drawing from sociological and social psychological theories of health‐related stigma (key examples include^7^, ^18^, ^36^, ^37^, ^38^, ^39^, ^40^). A preliminary summary of findings was provided to all research participants, with several providing constructive feedback on the content, organisation and interpretation of these findings.

## 3 RESULTS

The following sections provide an overview of the findings of the deliberative discussion, that is, what stigma‐reduction interventions (summarized in Table 2) were described by participants and how these interventions drew on tacit understandings of the stigma concept. In reading this presentation of results, it can be helpful to imagine deliberants navigating the stigma concept along two axes: one that draws attention to either disease attributes or personal (moral) attributes as the object of stigmatisation, and one that positions stigma as an individual or structural problem. The headings used in this section are used to illustrate movements around the poles of these conceptual axes.

**Table 2:** Suggested interventions for stigma‐reduction work relevant to persons with T2DM

Stigma focus	Specific targets for action	Potential actions (internal to diabetes organisations)	Potential actions (external to diabetes organisations)
Addressing the health problem	Support persons living with diabetes to efficaciously perform self‐management tasks and develop a positive disposition towards one's diabetes and its care requirements	Provision of individual healthcare services	Support engagement of persons with diabetes with individual healthcare services
	Support individual weight‐reduction	Provision of individual healthcare services	Support engagement of persons with diabetes with individual healthcare services
Addressing the emotional impact of stigmatisation	Enhance the ability of persons living with diabetes to cope with disease stigma	Psycho‐education (stand‐alone or integrated into existing counselling and disease self‐management interventions)	Encourage help‐seeking behaviour in support of individual counselling
Addressing the stigmatizers	Promoting a *factual* understanding of the aetiological complexity of diabetes	Organisational communications	Changing media representations of diabetes and persons with diabetes
	Clearly communicating the current status of knowledge regarding norms of self‐management for persons with diabetes	Organisational communications	Changing media representations of diabetes and persons with diabetes
	Raising the visibility of practices and discourses contributing to feelings of shame and/or guilt	Service codesign	Breaking silence through social‐justice‐oriented movements
Policy and advocacy work	Transformation of the way that persons with diabetes are represented—towards the socially embedded but capable agent	Service codesign Provision of individual healthcare services guided by principles of person‐centred care	Advocacy for person‐centred care

Abbreviation: T2DM, type 2 diabetes mellitus.

### 3.1 Focusing on ‘disease stigma’: Reducing stigma by addressing the health problem and correcting diabetes misinformation

Discussion under this heading is united by a belief that certain features of diabetes, or disease attributes, are central to the operation of diabetes‐related stigma.^7^, ^41^ This logic meant that stigma‐reduction work would logically involve either removing the stigmatized (disease) attribute by ‘addressing the health problem’^19^ or changing the way that people understand the attribute. In relation to addressing the health problem, a prominent narrative that featured within each group was the organisational imperative to ‘empower’ persons with diabetes to optimize management of their diabetes, something that was currently being addressed through the organisation's existing suite of individual and group education services. This approach assumed that affected individuals, to some extent, have a role to play in modifying stigmatized attributes of obesity (or fatness) or diabetes‐related complications. Although the notion of individual empowerment was widely accepted by participants as a desirable activity, one participant (HCS1) offered a critique of the empowerment concept (as used by other deliberants) by suggesting that it conceals the way in which personal agency, assumed to operate freely within the notion of empowerment, is constrained by what she referred to as ‘social determinants’, making generic reference to social determinants of health framework. This participant asserted that not everyone had the same capacity to deflect or circumvent stigmatisation through acts of self‐care and weight reduction.

Another group of actions, already occurring in a limited way via existing channels of communication, included attempts to communicate *factual* information about diabetes or correcting inaccurate information. Specifically, participants described how news media has tended to draw on obsolete understandings of diabetes as a ‘death sentence’, has oversimplified diabetes aetiology in a way that portrays T2DM as a self‐inflicted disease, and has established inaccurate ideas about the ‘diabetic diet’. For participants in this study (BRD/HCS1/2), diabetes organisations were seen to have a potential role of providing accurate information about diabetes to counter or ‘dilute’ inaccurate messages produced via news media. Participants proposed that a more accurate communication of information about diabetes could potentially be achieved through mass communication efforts, involving media liaisons or ambassadors, conducted independently of (i.e., initiated and performed by the diabetes organisation) or in collaboration with existing news‐media organisations (i.e., collaborative work initiated by and maintained by the diabetes organisation, but performed by media organisations). Member stories were seen as a powerful means of obtaining audience attention and communicating ‘factual’ medico‐scientific information about diabetes while generating positive attitudes towards persons with diabetes and their efforts towards self‐management (BRD/HCS/COM).

I think if you go down the media path and you have a media personality that might be about profile. But you really need a person that actually understands diabetes. And that's not just a health professional; they are people living with diabetes. So the approach that I'd like to see is that you actually have ambassadors that actually have diabetes across the types of diabetes, that are trained, are able to – and most people – you saw it in our TV commercials that we developed for our campaigns last year – we picked certain people, board included, that could actually talk about their personal experiences of living with diabetes. (BRD)

However, the assertion that diabetes organisations must provide factual and unembellished information about diabetes was met with the counter‐argument that such a neutral stance was difficult to achieve given the public health functions of the organisation. For example, several participants identified how an emphasis on obesity as a modifiable risk factor for T2DM development is instrumental in motivating individuals to promptly identify and manage health risks (BRD/COM/HCS1). Within a more formally expressed logic, one participant in the HCS1 group claimed that risk communication efforts need to be sufficiently ‘strong’ to promote behaviour change, justified using the theoretical logic of the Health Belief Model.^42^

### 3.2 Focusing on ‘symbolic stigma’: Reducing stigma by challenging moral beliefs about persons living with diabetes

Taking an explicitly neutral stance on communicating ‘facts’ about diabetes was challenged on the basis that stereotypes and prejudice often draw heavily on moral concepts about persons living with diabetes. Therefore, a distinction was drawn between scientific beliefs about diabetes per se and moral beliefs about those living with diabetes, which formed a type of ‘symbolic stigma’ whereby diabetes is seen as subjectively informative of irresponsible moral character.^41^ The disease and symbolic stigma distinction is evident in the following excerpt, which emerged in response to claims regarding the need to communicate more scientific‐factual information about diabetes and its management.

My initial reaction was it's a focus on diabetes from the standpoint of lifestyle exclusively, and in a way I think it's creating its own stigma with that kind of reporting and that kind of message rather than the cross section of all those people affected by diabetes. I think it creates a stigma of people don't look after themselves, don't manage their condition and that people that have diabetes are lose/wins [losers or winners], which, as I say, is not in all cases, but it creates this, I guess, stigma for me also, these people, why should they be helped, because they're creating a problem for themselves. (COM)

The idea of adopting a neutral communicative stance was also countered by the need to strategically portray life with diabetes in positive terms. For members of the board, the status quo is for diabetes to be framed in a way that is obstructive to living a good life, drawing on notions of suffering, and that the positive framing or normalizing of life with diabetes provides a means of counteracting unhelpful portrayals of life with diabetes (BRD). However, attempts to de‐emphasize the suffering of diabetes might also have the undesirable consequence of communicating a reduced need for research or support services for those living with diabetes (BRD) and might also fail to adequately recognize the challenges of diabetes self‐management, potentially running afoul of the organisation's goal of maintaining an empathetic relationship with members (HCS1/2). Following a similar logic to the HCS groups, board members also deliberated on the benefits of communicating the capabilities of persons with diabetes in an assets‐based manner (to offset the frequent problematisation of persons with diabetes) versus the risk that an assets‐based approach may further contribute to the blaming of those that have ‘failed' to effectively manage their diabetes, particularly in contexts where diabetes incidence and management is structured by social issues such as poverty.

### 3.3 Focusing on ‘self/felt stigma’: Reducing the adverse emotional impact of stigmatisation

Although no group identified existing actions to specifically reduce the adverse emotional effects of stigmatisation, the HCS1/2 and COM groups suggested that a potentially efficacious action would be to provide education to those with diabetes to support these individuals to better cope with stigmatisation. This was premised according to two lines of reasoning. First, it was argued that for those experiencing stigmatisation, the internalisation of stigmatizing beliefs must be disrupted with information that: (i) stimulates a self‐awareness of these internalized beliefs, where relevant, and (ii) brackets off cultural beliefs that are either not personally relevant or are otherwise unhelpful to the central task of diabetes self‐management. Second, a focus on education as a stigma‐reduction strategy was premized on the belief that dominant stereotypes about persons with diabetes are relatively stable within society, and that educational interventions act to emphasize personal agency in a context that functions to constrain it—both because of the effects of stigmatisation and because of broader paternalistic practices affecting those with diabetes. The following excerpt illustrates how this rationale converges within the notion of patient empowerment, which in this context offers a strategy for deflecting (cf. challenging) stigmatisation.^43^

If you have awareness campaign about preventing complications then you will actually learn that even when someone tells you it's really bad, you will know, well actually I have that knowledge, the powerful knowledge that I know that it's preventing complications that I have the power, I feel empowered to actually deal with the condition as it is. (COM)

### 3.4 Focusing on ‘structural stigma’: Reducing stigma through policy reform and advocacy

As an organisational work‐in‐progress and a broad aspirational statement, all groups within this deliberative research sought to establish their organisation as a highly visible, credible and authoritative voice in representing diabetes and persons living with diabetes. In developing a credible and authoritative voice, members of one group (HCS1) discussed the central role of service codesign policy, which was in the early stages of development within the organisation. In relation to stigma‐reduction work, codesign was described in a way that assumes that those living with diabetes are inherently capable of identifying and correcting stigmatizing practices contained within the activities and communications performed by the diabetes organisation, and in doing so can reduce the exposure of persons with diabetes to distressing content produced by the organisation. However, this assumption was also problematized within the group, based on observations of persons with diabetes contributing to a stigmatizing discourse by labelling themselves as ‘diabetics’. The idea of codesign was also problematized with reference to behaviour–change interventions (particularly those informed by the Health Belief Model) that require a certain level of (paternalistic) manipulation of cognitive and affective processes to motivate desirable behaviour. Therefore, there were limits placed on both the ability of persons with diabetes to identify stigmatizing practices and the organisation's ability to avoid these practices (if identified) given limits on autonomy in the face of a preventative health agenda:

P1: We are really sensitive and careful when we're developing content about, for example, any services or program in the future and it's a challenge for us to balance between raising awareness and in the meantime, protecting participants' mental health, in terms of not creating distress or a negative emotion as is it also our message, because message needs to be strong, because we know from the health belief model that we have to communicate and the more they are aware or they are concerned about the consequences of disease, the more they think with intent to change their behaviour. So that's for us, a challenge how to include in the design and delivery of services and programs.

P2: And that's where the co‐design can certainly come into it. (HCS1)

Although codesign was described with reference to actions taken internal to the organisation, there was further discussion about how the organisation might support healthcare reforms towards a model of person‐centred care (PCC). Across the deliberative groups, PCC was described as an approach that might mitigate stigmatisation via several mechanisms:

1. PCC functions to draw attention to nonbehavioural factors, which contribute to difficulties with diabetes self‐management, contribute to weight gain or inhibit weight reduction, potentially limiting attributions of personal blame (HCS2).
2. PCC functions to support recognition of the diversity of aetiology and needs amongst persons living with diabetes, which is useful in overcoming over‐generalisations about those with diabetes (COM).
3. There exists a power‐differential between healthcare providers and persons with diabetes, which allows for the reproduction of stigmatizing and blaming practices and discourses. PCC provides a mechanism for disrupting unequal modes of interaction (HCS1).

Implying that they themselves assume the role of the stigmatizer at times, several participants (COM/HCS1/2) argued for the need to reflect on one's own practices and identify how these practices might unintentionally contribute to the stigmatisation of those with diabetes. This need was justified given past observations of other diabetes organisations employing fear‐based tactics for purposes of fund‐raising (COM), and recognition of the way that the training of healthcare professionals has led to the uncritical adoption of assumptions about overweight persons and persons with chronic illness (HCS1/2). For the HCS1 group, several participants recognized the unequal power that tends to exist between healthcare providers and persons with diabetes, which means that the onus is placed on healthcare providers to reflect on the stigmatizing potential of taken‐for‐granted language and practices.

## 4 DISCUSSION

In interpreting these findings, it is apparent that participants drew from a complex assemblage of ideas about diabetes, persons with diabetes and stigmatisation. This is not unique to diabetes‐related stigma, but rather reflects the conceptual complexity observed in relation to other stigmatized conditions, such as with persons living with HIV–AIDS^7^ or mental illness.^18^ In the present study, lay understandings of the stigma concept tended to be cleaved in two ways: *disease* versus *symbolic* stigma and *individual* versus *structural* stigma. These cleavages are important because they shape the type of grievances raised and the consequent selection of stigma‐reduction interventions. Such grievances include misunderstandings of medical ‘facts’ about diabetes (disease stigma), misrepresentation of the characters of persons with diabetes (symbolic stigma), the inability of persons with diabetes to manage feelings of stigmatisation (self‐stigma), or features of healthcare and health news that systematically uphold stigmatizing knowledge about diabetes and persons with diabetes (structural stigma). These cleavages can also be observed within extant literature on diabetes‐related stigma, which predominantly focuses on self‐stigma and symbolic features of stigmatisation,^11^, ^14^, ^44^, ^45^, ^46^, ^47^, ^48^, ^49^, ^50^ with smaller literature examining the structural basis of stigmatisation.^51^, ^52^, ^53^ Although there are few examples of stigma‐reduction work relevant to diabetes, existing diabetes advocacy work^21^ and participatory action research^54^ reveals a focus on dispelling myths about diabetes, challenging misrepresentations of diabetes and persons with diabetes in media and health education texts, and improving the ability of individuals to care for their diabetes and cope with the adverse emotional effects of living with diabetes, following the cleavages observed in the present study.

What the present research adds is that stigma‐reduction efforts would likely benefit from addressing multiple grievances in ways that accommodate intervention at individual and structural levels while also focusing on attributes related to diabetes and persons with diabetes. It is likely that interventions to reduce the emotional effects of ‘internalised’ or ‘felt’ stigma are likely to be readily subsumed under key policy areas of reducing diabetes‐related distress and improving the mental health of persons living with diabetes.^55^, ^56^ While these individual‐based interventions are not in and of themselves problematic, stigma‐reduction work in relation to other stigmatized conditions has experienced movement towards addressing the structural causes of health‐related stigma, particularly when seeking to reduce stigma at a population scale.^5^, ^26^, ^38^, ^39^, ^40^, ^57^, ^58^ Given the paucity of literature examining the structural basis of diabetes‐related stigma (with some key exceptions)^51^, ^52^, ^53^ and the existence of conceptual barriers to thinking about stigma in structural ways,^58^ it would appear that stigma‐reduction work needs to actively seek to operate at *both* individual and structural levels. In this study, actions to challenge stigmatisation at a more structural level were apparent in references to PCC, service codesign and media advocacy work, following broader policy drivers towards public and patient involvement or ‘consumer engagement’ in healthcare and the democratisation of healthcare planning, implementation and evaluation.^59^, ^60^ Likewise, although a focus on both disease stigma and symbolic stigma is warranted given their synergistic effect on the experience of stigmatisation,^41^, ^61^ a focus on persons living with diabetes (cf. diabetes itself) is likely to draw greater attention to matters of identity, which is central within the stigma concept.^62^ Simply correcting misinformation about diabetes with medico‐scientific ‘facts’ may simply deflect blame away from those ‘normal’ persons with diabetes while doing little to challenge the production of stigmatized subgroups. While this conceptual map of stigma‐reduction interventions may prove useful in intervention planning and strategy, further interventional research is required to identify the effectiveness of these proposed interventions.

In interpreting results from the deliberative case study, key quality issues relate to how representative the sample was and to what extent findings can be generalized to other diabetes organisations.^24^, ^63^ Representativeness in this deliberative research refers to the ability of the research to access a diversity of opinion regarding diabetes‐related stigma and stigma‐reduction work. Although this study obtained views primarily from what Degeling et al.^64^ refer to as an *advocate* public (as an educated and partisan group), perspectives were not sampled from *affected* (persons living with diabetes and their families) and *lay* publics. While this focus is justified given our intention to examine the opinions of those likely to engage most directly with stigma‐reduction work, this may have missed important contributions by persons with diabetes who engage with stigma‐reduction and advocacy work peripheral to or outside of formal organisations.^46^ In relation to generalisability, it is recognized that different diabetes organisations will operate within different policy and cultural contexts and have access to different human, financial and informational resources, which will likely shape opinion regarding diabetes‐related stigma and viable approaches to stigma‐reduction work. However, cross‐cultural research has demonstrated that conceptual understandings of diabetes‐related stigma are relatively robust,^47^, ^51^ meaning that the findings from this study are also likely to have broad national and international relevance at a conceptual level.

## 5 CONCLUSION

Recognizing that existing stigma‐reduction work relevant to people with diabetes has been hampered by a lack of conceptual clarity around the stigma concept, this deliberative research sought to characterize how staff and board members within an Australian state‐wide diabetes organisation currently conceptualize the problem of diabetes‐related stigma and how these conceptual understandings are used to justify existing stigma‐reduction work or envisage future approaches to stigma‐reduction. Findings from this study suggest that stigma‐reduction interventions take their form given assumptions about the object of stigmatisation (i.e., the disease vs. persons living with the disease) and the location of stigmatizing processes (i.e., at an individual or structural level). While it may be simpler to conceptualize and act on diabetes‐related stigma as an individual‐level phenomenon or a community‐level phenomenon involving misunderstandings about diabetes, engaging with structural and symbolic understandings of diabetes‐related stigma will help ensure that stigma‐reduction work engages with existing theorizing about diabetes and health‐related stigma in a comprehensive way.

## CONFLICT OF INTERESTS

The diabetes organisation examined in this research provided funding to the authors to perform this research. As an ethical requirement, participants were offered opportunities to review and suggest amendments to the content, organisation, and interpretation of these findings.

## Data Availability

The data that support the findings of this study are available on request from the corresponding author. The data are not publicly available due to privacy or ethical restrictions.
